# A Novel Peptide from Soybean Protein Isolate Significantly Enhances Resistance of the Organism under Oxidative Stress

**DOI:** 10.1371/journal.pone.0159938

**Published:** 2016-07-25

**Authors:** Heran Ma, Rui Liu, Ziyuan Zhao, Zhixian Zhang, Yue Cao, Yudan Ma, Yi Guo, Li Xu

**Affiliations:** 1 Key laboratory for Molecular Enzymology and Engineering, the Ministry of Education, National Engineering Laboratory for AIDS Vaccine, School of Life Sciences, Jilin University, Changchun, Jilin, 130012, P.R. China; 2 Department of endocrinology, China-Japan Union Hospital, Jilin University, Changchun, Jilin, 130033, P.R. China; 3 Sports Science Research Institute of Jilin Province, Changchun, Jilin, 130022, P. R. China; University of Pecs Medical School, HUNGARY

## Abstract

Recent studies have indicated that protein hydrolysates have broad biological effects. In the current study we describe a novel antioxidative peptide, FDPAL, from soybean protein isolate (SPI). The aim of this study was to purify and characterize an antioxidative peptide from SPI and determine its antioxidative mechanism. LC–MS/MS was used to isolate and identify the peptide from SPI. The sequence of the peptide was determined to be Phe-Asp-Pro-Ala-Leu (FDPAL, 561 Da). FDPAL can cause significant enhancement of resistance to oxidative stress both in cells as well as simple organisms. In *Caenorhabditis elegans (C*. *elegans)*, FDPAL can up-regulate the expression of certain genes associated with resistance. The antioxidant activity of this peptide can be attributed to the presence of a specific amino acid sequence. Results from our work suggest that FDPAL can facilitate potential applications of proteins carrying this sequence in the nutraceutical, bioactive material and clinical medicine areas, as well as in cosmetics and health care products.

## Introduction

According to the free radical theory proposed by Denham Harman, living organisms can produce radical species by various metabolic pathways. These include reactive oxygen species (ROS), such as O_2_^-^, HO_2_, H_2_O_2_ and OH [1]. Excessive accumulation of free radicals, known as oxidative stress, can be harmful to the cell. Usually, oxidative stress is considered to be a promoter of chronic diseases, such as cancer, diabetes, Alzheimer’s disease and others, rather than an initiator [2–5]. Extensive investigations have indicated that dietary supplementation of antioxidants can enhance the body’s natural defense mechanism. This appears to be a reasonable and practical approach to reduce the level of oxidative stress *in vivo* [6, 7]. Over the past several decades, a large number of natural antioxidants have been obtained from animal, plant, and even microbial sources [8–12].

For many years, Soy products have been one of the main sources of dietary protein, and it is therefore of relevance to investigate the presence of any potential additional bioactivity that can meet the need to counter the increasingly higher incidence of environmental stress. Normally, enzymatic hydrolysis or fermentation is used to enhance the functionality of protein ingredients, which may lead to the production of short peptide sequences with various bioactivities. After enzymatic hydrolysis, the resulting lower molecular mass of peptides will likely have more potential to exert biological effects *in vivo* due to their increased permeability through the intestinal cells [13, 14]. Soybean protein hydrolysates that possess a range of bioactivities including antioxidation, anti-hypertension, anti-hyperlipidemia, cholesterol reduction and immunity enhancing activities have been extensively reported in literature [15–18].

In this research we propose the hypothesis that peptides from soybean protein possess antioxidant activity and that this activity contributes to its various bioactivities. However, we cannot investigate the antioxidation mechanism of these peptides unless we determine their precise amino acid sequences. Liquid chromatography tandem mass spectrometry (LC–MS/MS) is the preferred method for separation and identification of peptides in complex bioactive peptide mixtures. In this article, we purified and identified peptide sequences using continuous chromatography and LC-MS/MS methods. We identified a purified peptide with the sequence Phe-Asp-Pro-Ala-Leu (FDPAL, 561 Da) from soybean protein isolate (SPI). The antioxidant activities of FDPAL were subsequently evaluated, and were found to include scavenging free radicals *in vitro*, reducing the accumulation of ROS and lipofuscin, up-regulating the expression of SOD-3 *in vivo*, improving the survival rate of Hela cell and *C*. *elegans* under oxidative stress. We conclude that the activity of this pentapeptide is related to its amino acid composition and sequence.

The specific objectives of this study were to: (i) isolate the antioxidant peptide from SPI and determine its primary structure; (ii) evaluate the antioxidant activities of this peptide both *in vitro* and *in vivo*; (iii) elucidate its antioxidation mechanism at molecular and cellular levels, and also in multicellular organisms, and subsequently to determine whether these mechanisms are consistent with each other.

## Materials and Methods

### Materials

Soybean protein isolate (SPI) was purchased from Daqing Celestial Sun Moon Star Protein Co., Ltd., China. An enzyme (Alcalase) was purchased from Novo Nordisk, Shenyang Biochemical Processing Co. Ltd., China, which was used in enzymolysis. Sephadex G-10 and DEAE Sephadex A-25 were purchased from Pharmacia Chemicals Co., Sweden. All other reagents used were of analytical grade and obtained from Sigma Chemical Co, USA.

### Enzyme hydrolysis

10 g SPI was added to 100 mL distilled water in a 250 mL conical flask. The flask was then incubated in a water-bath at a temperature of 50°C, and the pH of the solution was adjusted to 8.0. After hydrolysis was allowed to occur for the required length of time, the conical flask was placed in a boiling water bath and incubated for 10 min in order to inactivate the enzyme and terminate the enzymatic reaction. The aqueous solution was then rapidly cooled to room temperature and centrifuged at 7000 rpm for 15 min. The supernatant was removed till only 30 mL remained. The degree of hydrolysis (DH) was determined using the pH stat method based on the Eq (1) [19]:

$$\text{DH}=\text{B}\times \frac{{\text{N}}_{\text{b}}}{\alpha \times {\text{M}}_{\text{p}}\times {\text{h}}_{\text{tot}}}\times 100\%$$ (1)

where B is the volume of NaOH in the hydrolysis process (mL), N_b_ is the normality of NaOH (mol/L), M_p_ is the mass of protein used (g), h_tot_ is the amount of peptide bonds per gram of SPI (7.80 mmol/g) [20], α is the degree of dissociation of α-amino and determined by Eq (2):

$$\alpha =\frac{10^{\text{pH}-{\text{pK}}_{\text{a}}}}{1+10^{\text{pH}-{\text{pK}}_{\text{a}}}}$$ (2)

where pH is the initial pH of enzymatic hydrolysis (8.0) and pK_a_ is a dissociation constant, which is calculated by Eq (3):

$${\text{pK}}_{\text{a}}=7.8+\left(\frac{298-\text{T}}{298\times \text{T}}\right)\times 2400$$ (3)

where T is the temperature of enzymatic hydrolysis (K).

### Ion-exchange chromatography

DEAE Sephadex Fast Flow ion-exchange column (2.0 cm × 20 cm; Pharmacia Fine Chemicals, Sweden) was used to separate the components of the lyophilized SPI powder with 10 mM phosphate buffer (pH 9.0). The column was previously equilibrated with buffer until no absorbance peaks were observed and then a linear gradient elution of 0–5 M NaCl was performed at a flow rate of 1 mL/min. The eluate was collected and evaluated at 220 nm. Fractions (fractions 1–3) with distinct peaks were collected and lyophilized to test for antioxidant activity (S1A and S1B Fig).

### Gel filtration chromatography

As shown in S1A and S1B Fig, fraction 2 was found to have the highest antioxidant activity following ion-exchange chromatography. This fraction was dissolved in distilled water and loaded onto a Sephadex G-10 gel filtration column (2.0 cm × 100 cm; Pharmacia Fine Chemicals, Sweden) and eluted with distilled water at a flow rate of 0.5 mL/min. The eluate was collected and evaluated at 220 nm. Fractions with distinct peaks were collected and lyophilized to test for antioxidant activity (S1C and S1D Fig).

### Determination of amino acid sequence by LC-MS/MS

The LC-MS/MS system consisted of an Eksigent Ekspert UltraLC100 Reversed Phase High Performance Liquid Chromatography (RP-HPLC) system coupled to an AB SCIEX Triple TOF™ 5600 mass spectrometer with Turbo spray. The chromatography column was a reverse phase C18 column, (serial No. 186003023, 3.5 μm, 2.1 mm × 150 mm Waters XBridge). 10 μL of sample was injected and subsequently eluted with a gradient elution program, with a linear gradient of 0.1% methanoic acid in distilled water (A) and 0.1% methanoic acid in acetonitrile (B) from 10% B to 90% B in 15 min, at a flow rate of 0.25 mL/min. The scan range was set at m/z 100.00–1000.00. The MS/MS spectrum was sequenced by manual calculation and pure peptide FDPAL was obtained.

### Fenton’s reaction

Hydroxyl free radicals generated by Fenton’s reaction were measured by monitoring chemiluminescence of the reaction mixture. Reagents were added into a cuvette in the following order: 10 μL 3% H_2_O_2_, 10 μL 0.1 mM Fe^2+^, 10 μL FDPAL, and 970 μL 0.1 mM luminol (in sodium carbonate buffer, pH 10.2), and were incubated in a water bath at 25°C. A series of reactions with a final FDPAL concentration 0.05, 0.1, 0.2, 1 and 2 mM were set up and absorbance was measured at 550 nm. Vitamin C (Vc) group was treated as control.

### Pyrogallol self-oxidation assay

The *in vitro* superoxide anion-scavenging characteristic of FDPAL was measured using a pyrogallol autoxidation system with modifications [21]. Reagents were added into a cuvette in the following order: 10 μL 3 mM pyrogallol, 80 μL 4 mM NaOH, 10 μL FDPAL, and 900 μL 0.1 mM luminol (in sodium carbonate buffer, pH 10.2) and incubated in a water bath at 25°C. A series of reactions with a final FDPAL concentration of 0.05, 0.1, 0.2, 1 and 2 mM were set up and absorbance was measured at 325 nm. Vitamin C (Vc) group was treated as control.

### MTT assay

HeLa cells were purchased from China General Microbiological Culture Collection Center (CGMCC, Beijing, CHN), and were grown exponentially for investigation. The cells were seeded in 96-well plates at a final concentration of 8 × 10^3^ cells per well and incubated with FDPAL at various concentrations (0, 0.1, 0.2, 1, 2, 10, 20 mM) for 12 h prior to the addition of 500 μM H_2_O_2_. The medium was then removed and the cells were washed twice with PBS. Fresh low serum (5%) medium containing 500 μM H_2_O_2_, was then added to the cells and incubated at 37°C, 5% CO_2_ for 4 h. At the indicated time, MTT assay was used to evaluate the cell survival rate [22].

### Worm strains and their maintenance

*C*. *elegans* was grown in a standard nematode growth medium (NGM) in plates maintained at 20°C and fed with live *Escherichia coli* OP50 bacteria (Brenner 1974). The wild-type strain Bristol N2 and the transgenic strain CF1553 (muIs84) were obtained from Caenorhabditis Genetics Center; CGC, USA. SOD-3::GFP-linked reporter in CF1553 was used to visualize SOD-3 expression.

### Stress resistance assay

Stress resistance assay was performed with two-day-old adult worms. The worms were incubated for two days with FDPAL (10 mM) and were then transferred to plates with 500 μM juglone. Worm deaths per hour were counted and recorded. Each assay was performed in three independent parallel experiments in a double-blind manner [23].

### Measurement of intracellular ROS in *C*. *elegans*

For the ROS test under oxidative stress, two-day-old adult worms were treated with 300 μM juglone for 1 h and were then transferred to plates with or without FDPAL (10 mM). After two days of incubation, worms were transferred into a 96-well plate with a transparent bottom and opaque walls containing M9 buffer in the wells. A fluorescent probe (H_2_DCF-DA) was employed to determine the intracellular ROS in worms [24]. Fluorescence intensity correlated with the ROS level and was measured by a microplate reader at excitation and emission wavelengths of 485 and 520 nm respectively.

### Fluorescence quantification and visualization

Net fluorescence of worms was assayed using a Thermo Labsystems Fluoroskan Ascent microplate reader (Thermo Fisher, Waltham, MA). Adult worms were treated with or without 10 mM FDPAL for two days. 20 control or treated adult animals of the indicated age were transferred into each well of a Costar 96-well microtitre plate (black, clear, flat-bottom wells) containing 100 μL of M9 buffer, after which GFP and ROS fluorescence were measured using 485 nm excitation and 530 nm emission filters. Under ultraviolet light, gut granules emit blue fluorescence, which has been attributed to lipofuscin, with maximal intensity at λ_ex_/λ_em_ 340/430 nm. Four replicates were used for each determination. For fluorescence microscopy, the worms were suspended in a drop of levamisole (10 mM) and mounted on a cover slip layered with 3% agarose. The fluorescent images of worms were captured using an AXIO Imager M2 microscope system.

### Statistical analysis

Values were expressed as mean ± S.D. The statistical significance between six groups was evaluated by one-way ANOVA followed by Students-'t' test. Results with P-values less than 0.05 were considered to be significant (Graph Pad, San Diego, CA).

## Results

### Isolation and purification

**Fig 1:**
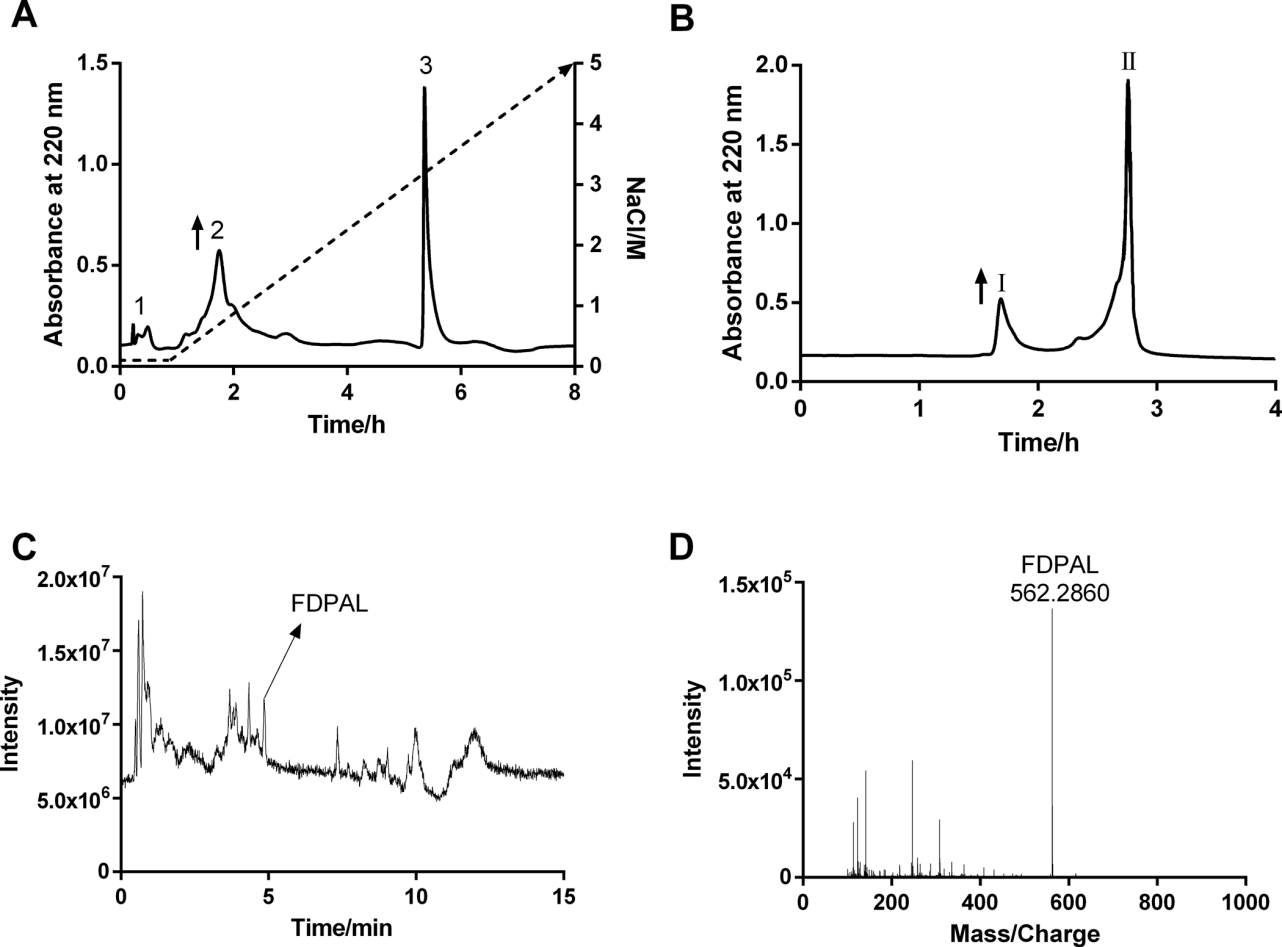
Purification and identification of FDPAL from Soybean Protein Isolate extraction. (A) Separation of antioxidant peptides from SPI by using Sephadex A-25. Elutions were performed with 10 mM phosphate buffer (pH 9.0) and a linear gradient of 0–5 M NaCl at a flow rate of 1.0 mL/min for 8 h. (B) Separation of fraction 2 in (A) by Sephadex G-10. Elutions were performed with distilled water at a flow rate of 0.5 mL/min for 4 h. (C) TIC spectrum of active fraction I from (B) measured by LC-MS/MS. (D) TOF-MS spectrum of FDPAL.

In our study, the enzymatic reactions were terminated when the DH values reached 24.64 ± 1.77%. DEAE Sephadex A-25 ion-exchange chromatography was then used to separate the SPI extract into three fractions (fraction 1–3). Fraction 2, which exhibited the strongest antioxidant activity (Fig 1A and S1A and S1B Fig) was further separated by Sephadex G-10 gel filtration column into two fractions (fractions I, II). Of these two, fraction I showed a strong antioxidant activity (Fig 1B and S1C and S1D Fig). LC-MS/MS was used to further purify and identify the monomer peptide from fraction I. We first eluted fraction I from a low organic mobile phase (10% acetonitrile) to a high organic mobile phase (90% acetonitrile), following which the monomer peptide FDPAL was purified from it. Triple TOF-MS was employed to determine the molecular weight of FDPAL, which revealed the molecular ion mass (M+H^+^) of FDPAL to be 562.2860 Da (Fig 1C and 1D).

### Characterization of purified peptides

**Fig 2:**
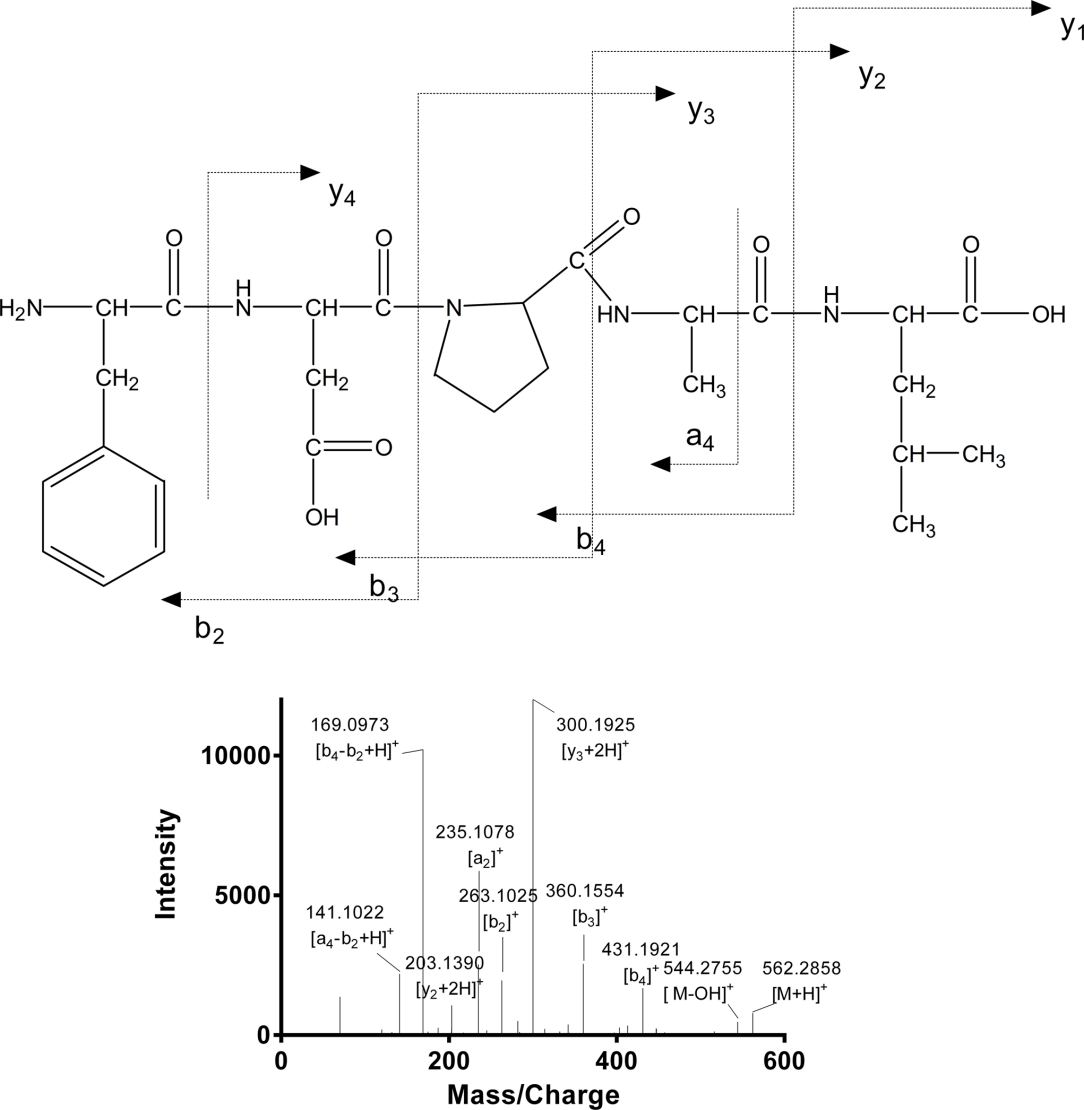
Identification of molecular mass and amino acid sequence of FDPAL. MS/MS experiments were performed on AB SCIEX TripleTOF™ 5600 mass spectrometer. The sequence of FDPAL was determined manually.

In order to identify the putative active peptide, an MS/MS experiment was performed on an AB SCIEX Triple TOF™ 5600 mass spectrometer. Fragmentation spectra that matched with the FDPAL fragment were sequenced manually. As shown in Fig 2, purified peptide FDPAL was identified to be a pentapeptide with the amino acid composition of Phe-Asp-Pro-Ala-Leu (561 Da).

### Antioxidant activity of FDPAL

**Fig 3:**
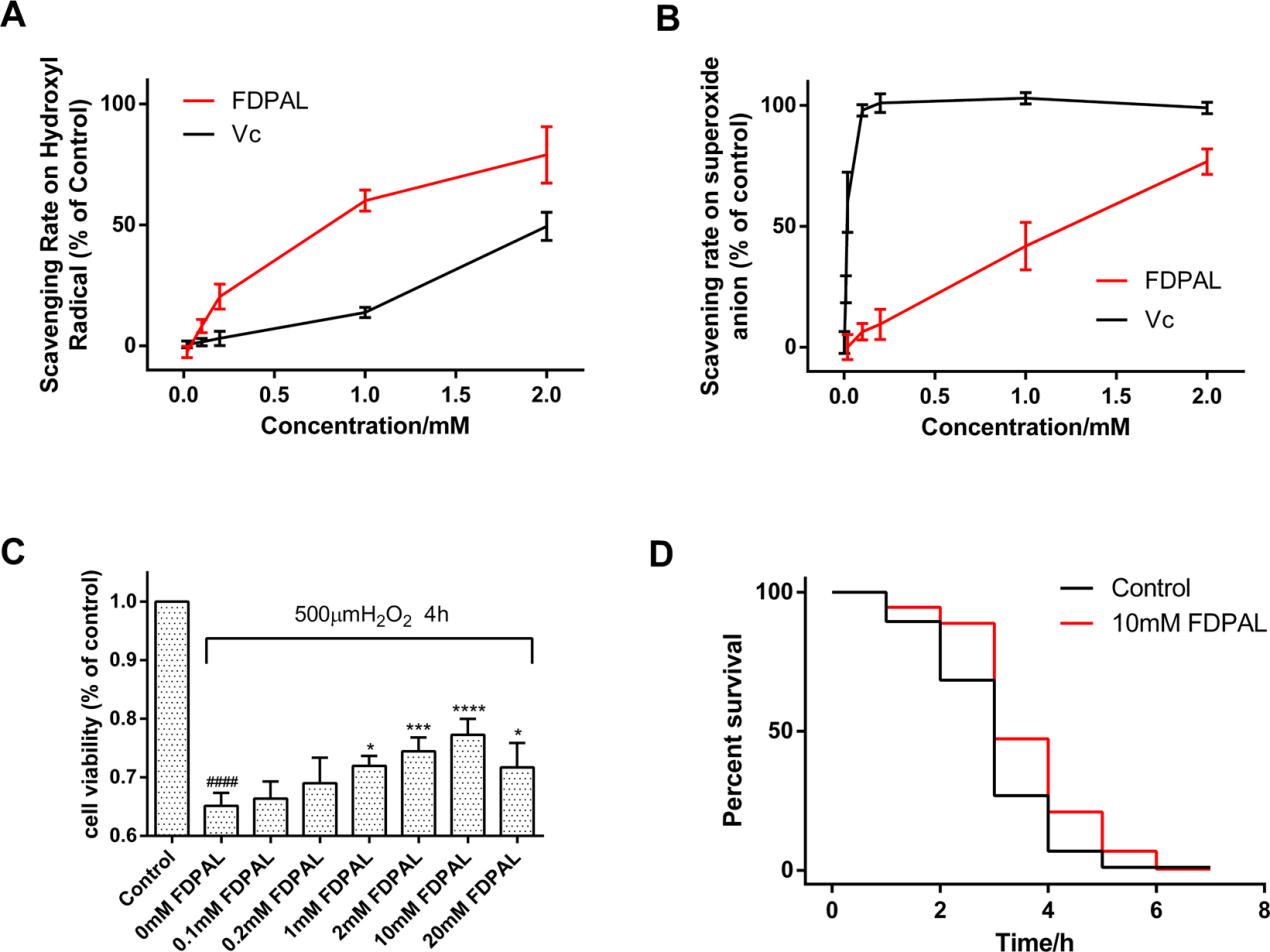
Antioxidant effect of FDPAL *in vitro* and *in vivo*. Vc group was treated as control. (A) FDPAL dramatically scavenged the hydroxyl free radical generated by Fenton’s reaction; n = 5 in each group. (B) FDPAL effectively scavenged the free radicals generated by pyrogallol self-oxidation; n = 5 in each group. (C) HeLa cell viability under oxidative stress with or without FDPAL. Significance of difference versus control group at ####p < 0.0001. Significance of differences versus 0 mM FDPAL group at *p < 0.05, ***p < 0.001, ****p < 0.0001; n = 5 in each group. (D) FDPAL improves the stress resistance of *C*. *elegans* under oxidative stress. Survival curves are presented based on three individual experiments.

Free-radical scavenging capacity of FDPAL was evaluated in subsequent experiments with the Vc group as control. Initially, the ability of FDPAL to remove hydroxyl free radical was investigated in the Fenton’s reaction system. The data revealed that FDPAL had a stronger effect of scavenging the hydroxyl free radical as compared to Vc (Fig 3A). Subsequently, the ability of FDPAL to remove superoxide anions was then investigated in a pyrogallol self-oxidation system. These data showed that FDPAL could also effectively scavenge the free radicals produced by pyrogallol self-oxidation (Fig 3B).

The MTT assay was used to assess the protective effect of FDPAL on H_2_O_2_-induced injury in HeLa cells. As shown in Fig 3C, after exposure of HeLa cells to 500 μM H_2_O_2_ for 4 h, the cell survival rate in the FDPAL groups increased significantly when compared with the control group. Preincubation of the HeLa cell with 0, 0.1, 0.2, 1, 2, 10 or 20 mM of FDPAL inhibited apoptosis. As shown in the results, FDPAL could significantly increase cell viability up to a concentration of 10 mM.

Further experiments were performed to evaluate the potential longevity-promoting effect of FDPAL on wild-type *C*. *elegans* N2 under oxidative stress. Worms that had just reached adulthood were pretreated with 10mM FDPAL for 48 h and then exposed to juglone (500 μM). Juglone is a pro-oxidant that can be reduced by diaphorases in the presence of NAD(P)H. It converts oxygen to superoxide anion and consequently increases intracellular oxidative stress. Our results showed that pretreatment with 10mM FDPAL had a strong protective effect. As shown by the statistical analyses, the mean survival rate was significantly increased by 24.2% in the FDPAL-treated group as compared to that of control (Fig 3D). The results indicated that FDPAL pretreatment improved resistance towards juglone induced oxidative stress in the worms.

**Fig 4:**
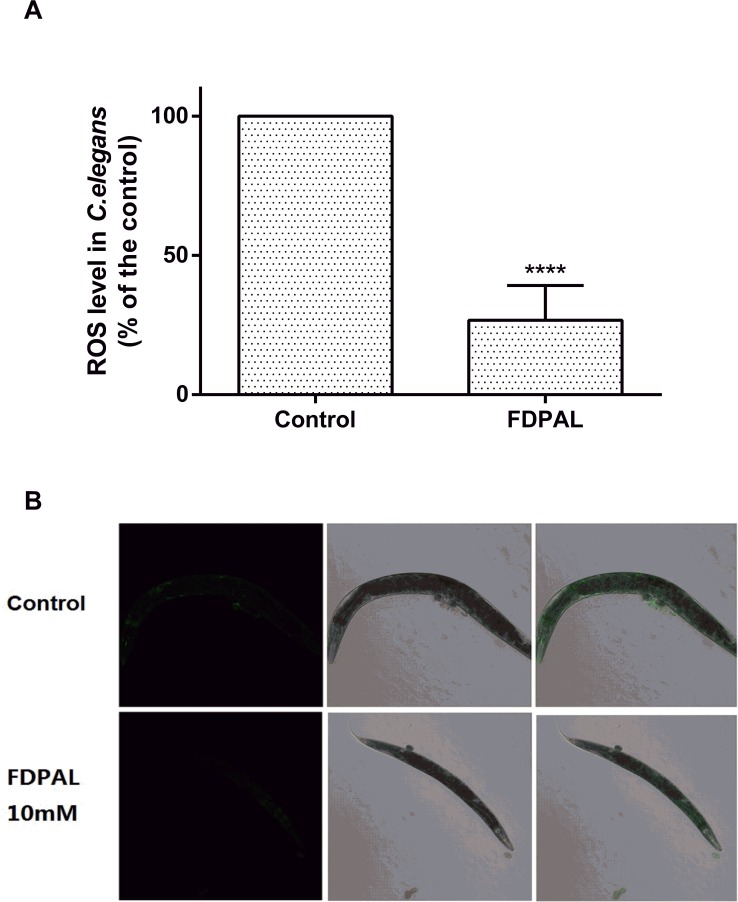
FDPAL at 10 mM reduced ROS accumulation in *C*. *elegans* N2 under juglone-generated oxidative stress. (A) Quantified DCF-DA intensity (±SE) in worms from three individual experiments with 50 worms per treatment (****p < 0.0001). (B) Image of fluorescence intensity in control worms and 10 mM FDPAL-treated worms. The ROS level in control worms is higher than that in FDPAL treated worms.

Additionally, FDPAL was also demonstrated to have ROS-scavenging capability in *C*. *elegans*. Our data showed that pretreatment of the worms with 10 mM FDPAL effectively reduced ROS accumulation in juglone-treated (300 μM) wild-type *C*. *elegans* (compared with the control, ****p < 0.0001; Fig 4).

These results suggested that FDPAL was a versatile free radical scavenger both *in vitro* as well as *in vivo*.

### FDPAL up-regulates SOD-3::GFP expression in transgenic *C*. *elegans* CF1553

**Fig 5:**
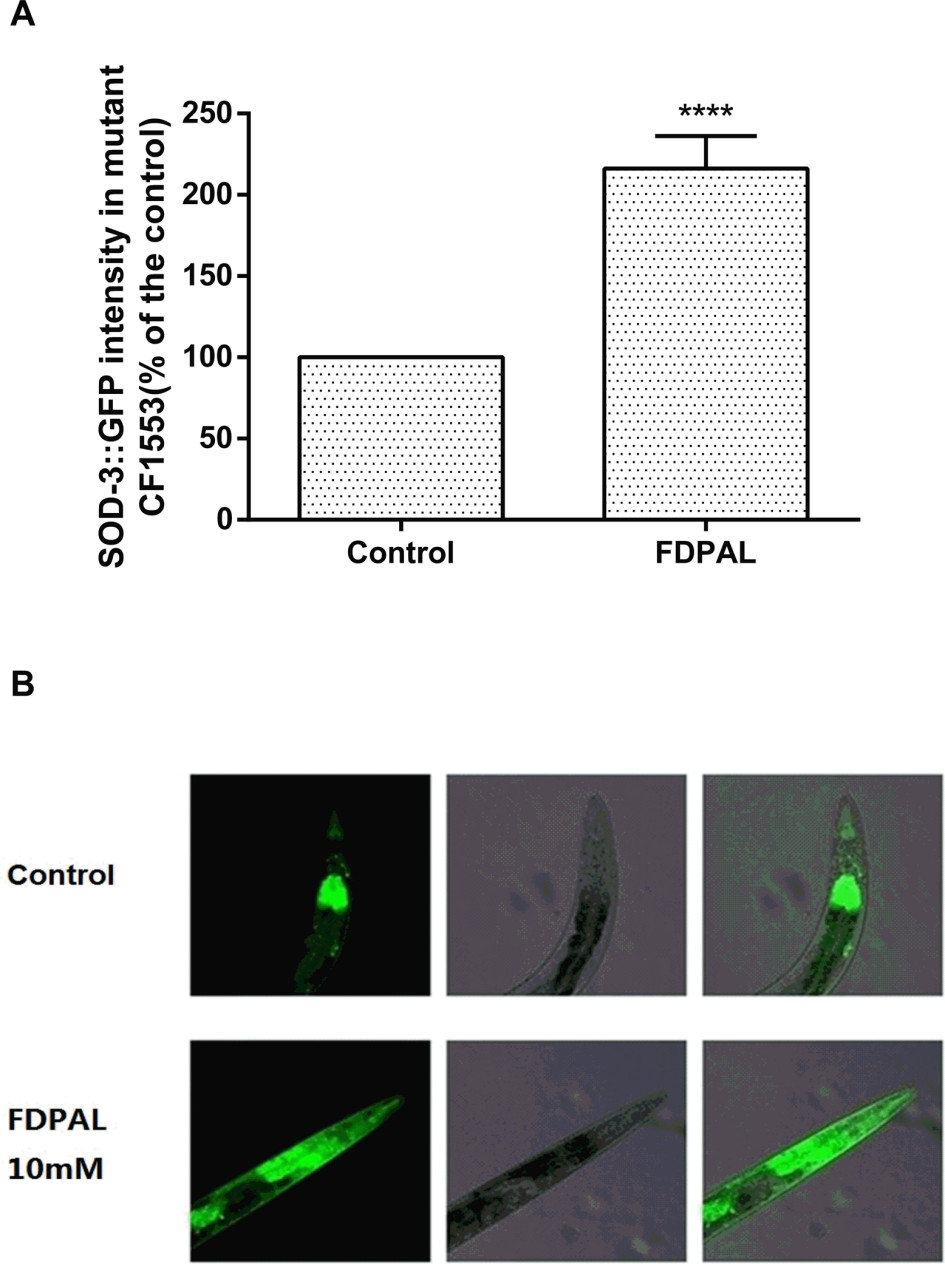
FDPAL up-regulates SOD-3::GFP expression in transgenic *C*. *elegans* CF1553. (A) Quantified GFP intensity (±SE) in CF1553 from three individual experiments with 50 worms per treatment (****p < 0.0001). (B) SOD-3::GFP expression in control worms and 10 mM FDPAL-treated worms. The SOD-3::GFP expression in FDPAL treated worms is higher than that in control worms.

To further investigate the mechanisms of the protective effects of FDPAL in *C*. *elegans*, SOD-3::GFP reporter gene expression in transgenic CF1553 was analyzed. The CF1553 worms were treated with FDPAL or left untreated after stimulation with juglone (300 μM). When compared with the control group, the FDPAL-treated group demonstrated a higher SOD-3::GFP intensity as observed with confocal laser scanning microscopy (Fig 5B). The fluorescence intensity was then quantified by a Thermo Labsystems Fluoroskan Ascent microplate reader. The results showed that 10mM FDPAL up-regulated SOD-3::GFP expression by 116.06% in transgenic CF1553 (Fig 5A), indicating that FDPAL could enhance the expression of SOD-3 under oxidative stress in *C*. *elegans*.

## Discussion

Traditionally, dietary protein is regarded as a potential source of energy and essential amino acids for humans and animals and has been the subject of numerous investigations. In recent times there is a growing interest in identifying antioxidative peptides from natural sources, including plants and animals [11, 25, 26]. These peptides may possess various properties, such as antioxidative [27, 28], antithrombotic [29], antimicrobial [30], immunomodulatory [31], opiate-like [32], mineral binding [33], hypocholesterolaemic [34] and antihypertensive [35, 36], owing to their structure, and amino acid composition and sequence. In this study, we purified a novel active peptide from SPI by chromatographic methods. The amino acid sequence of this peptide was determined to be Phe-Asp-Pro-Ala-Leu by AB SCIEX Triple TOF™ 5600 mass spectrometer (Figs 1C, 1D and 2). This novel mass spectrometer provides excellent MS performance combining high resolution, good mass accuracy and high sensitivity in order to achieve a high rate of success for identification.

We performed *in vitro* experiments to determine the antioxidant potential of this peptide. The results reveal that it possesses a high antioxidant efficacy (Fig 3), especially in the hydroxyl free radical scavenging test, where FDPAL was better in scavenging hydroxyl radicals when compared to Vc (Fig 3A). To further test whether FDPAL is permeable across the cell membrane and can exert biological effects, an MTT assay was designed. The H_2_O_2_-induced HeLa cells injury model was chosen for this assay to evaluate the protective action of FDPAL against acute oxidative injury [37, 38]. H_2_O_2_ was used as an inducing agent to mimic oxidative stress induced injury. As expected, exposure of cells to H_2_O_2_ resulted in decreased cell viability, an effect that was attenuated by FDPAL in a concentration dependent manner. Interestingly, we noticed a distinct drop in the surviving fraction when the concentration reached 20 mM (Fig 3C). This could be more acidic in 20 mM solution will injure cell, due to the presence of carboxyl group in FDPAL.

**Fig 6:**
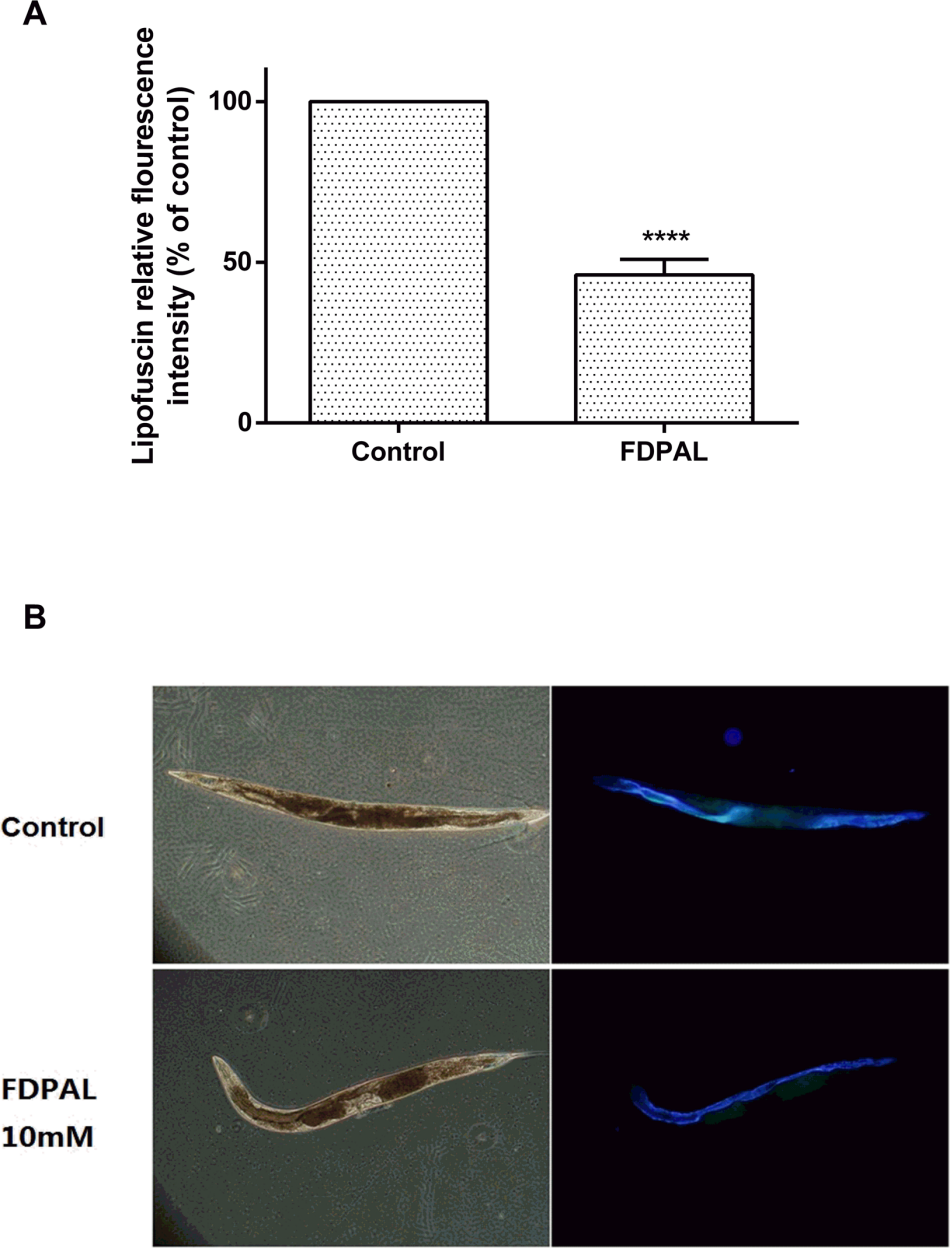
FDPAL can significantly reduce lipofuscin content in worms. (A) Lipofuscin fluorescence was measured by Image J, 30 worms were tested in each group. (B) Lipofuscin fluorescence in worms.

Thus we showed that FDPAL possesses a strong antioxidant activity *in vitro*. In order to test whether it has similar activity *in vivo*, we used *Caenorhabditis elegans* to investigate antioxidant activity *in vivo* under both normal and stress conditions. We found that FDPAL could significantly extend the life expectancy of *C*. *elegans* under oxidative stress conditions (Fig 3D), and reduce ROS and lipofuscin accumulation under normal conditions (Figs 4 and 6). We believe that this activity of FDPAL *in vivo* is related to its molecular weight, amino acid composition and sequence. An obvious drawback of protein and polypeptide drugs is their lower oral bioavailability due to enzymatic degradation in the gastrointenstinal tract and the impenetrable barrier of the intestinal epithelium. Generally in the gastrointestinal tract, most of the protein and polypeptide drugs are degraded to oligopeptides (2–6 amino acid residues) rather than free amino acids [39]. Thus FDPAL, due to its small size and low molecular weight (562 Da), can easily cross the intestinal barrier, thereby exerting its biological effects [40, 41]. In addition, the presence of hydrophobic groups at both the C-terminus (Leu) and N-terminus (Phe) renders FDPAL more soluble in lipids. This special feature of hydrophobic regions on both sides can also increase its affinity and reactivity to the intestinal cell membrane [6, 42]. In FDPAL, Phe-Asp are present as direct proton-donators, which, owing to their ability to quench unpaired electrons or radicals by supporting protons, are very important for the radical-scavenging activity of this peptide [43, 44]. Nonetheless, we believe that the activity of FDPAL does not completely depend on its own direct antioxidant capacity or its indirect oxidation resistance. We speculate that FDPAL influences the expression of genes associated with resistance in *C*. *elegans* and thereby enhances oxidative stress resistance. Our work indicates that FDPAL increases the expression of SOD-3::GFP under oxidative stress conditions in *C*. *elegans* (Fig 5). We speculate that FDPAL may activate certain regulatory pathways conferring resistance (such as the well-known insulin signaling pathway) in *C*. *elegans*, which then causes increased expression of SOD-3 [45, 46]. However, the exact mechanism by which FDPAL up-regulates SOD-3 expression is unclear, and we do not know yet whether FDPAL is released by enzymatic hydrolysis *in vivo* or not. These issues need further investigation to be resolved.

In summary, we present a novel antioxidant peptide, FDPAL, from SPI. The presence of specific amino acids and their sequence is responsible for the antioxidant activity of this peptide. Moreover, we found a longevity-promoting effect of FDPAL in *C*. *elegans* under oxidative stress conditions, which might be attributed to its direct ROS-scavenging activity and indirect free radical-scavenging activity, via up-regulation of expression of resistance associated genes such as SOD-3. In this study we describe, from the perspective of antioxidant activity, a series of influences that FDPAL has on organisms. Above all, these interesting findings suggest that FDPAL is an important peptide having potential applications in the nutraceutical, bioactive material and Clinical Medicine areas, as well as in cosmetics and health care products.

## Supporting Information

S1 Fig Antioxidant activities of the fractions in vitro. (A) Scavenging activities of fractions 1, 2 and 3 on superoxide anion. (B) Scavenging activities of fractions 1, 2 and 3 on hydroxyl radical. (C) Scavenging activities of fractions I and II on superoxide anion. (D) Scavenging activities of fractions I and II on hydroxyl radical.
(TIF)
