# Supplementary figures and images for: A Novel Peptide from Soybean Protein Isolate Significantly Enhances Resistance of the Organism under Oxidative Stress
Source: PLoS One. 2016 Jul 25;11(7):e0159938. doi: 10.1371/journal.pone.0159938 (PMC4959706; doi:10.1371/journal.pone.0159938)

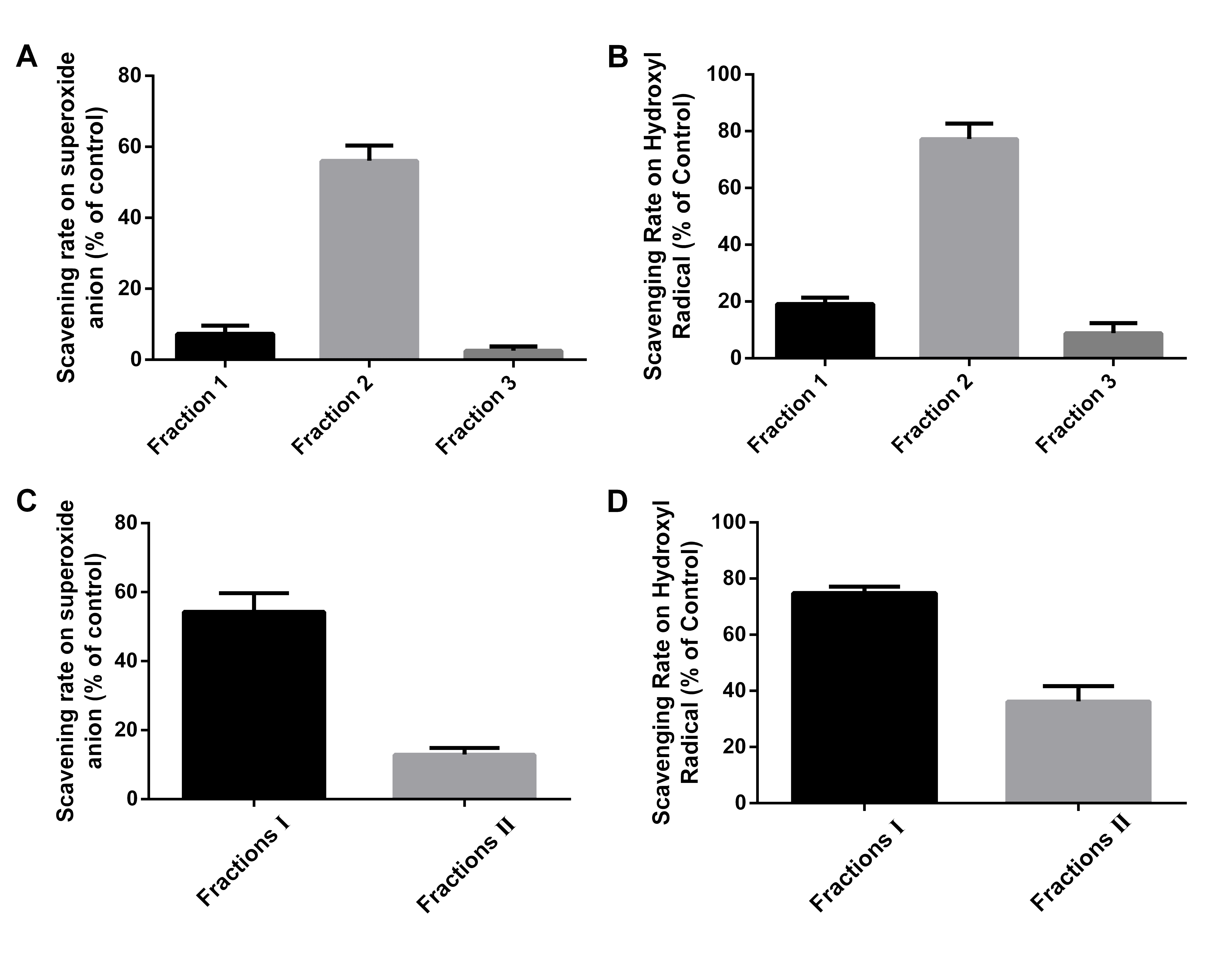

Supplement: S1 Fig — (A) Scavenging activities of fractions 1, 2 and 3 on superoxide anion. (B) Scavenging activities of fractions 1, 2 and 3 on hydroxyl radical. (C) Scavenging activities of fractions I and II on superoxide anion. (D) Scavenging activities of fractions I and II on hydroxyl radical. (TIF) [file pone.0159938.s001.tif]
